# Effect of Menstrual Cycle Phase on the Recovery Process of High-Intensity Interval Exercise—A Cross-Sectional Observational Study

**DOI:** 10.3390/ijerph20043266

**Published:** 2023-02-13

**Authors:** Pedro J. Benito, Víctor M. Alfaro-Magallanes, Beatriz Rael, Eliane A. Castro, Nuria Romero-Parra, Miguel A. Rojo-Tirado, Ana B. Peinado

**Affiliations:** 1 LFE Research Group, Department of Health and Human Performance, Faculty of Physical Activity and Sport Sciences (INEF), Universidad Politécnica de Madrid (UPM), 28040 Madrid, Spain; 2 Department of Physical Education, São Paulo State University (UNESP), Bauru 17033-360, SP, Brazil; 3 Departamento de Fisioterapia, Terapia Ocupacional, Rehabilitación y Medicina Física, Facultad de CC. de la Salud, Universidad Rey Juan Carlos, 28933 Madrid, Spain

**Keywords:** eumenorrheic, menstrual cycle, athletes, high intensity interval exercise, recovery

## Abstract

Although the study of the menstrual cycle influence on endurance exercise has recently increased, there is a lack of literature studying its influence on females’ cardiorespiratory recovery. Thus, the aim of the present work was to assess menstrual cycle influence on post-exercise recovery following a high intensity interval exercise in trained females. Thirteen eumenorrheic endurance-trained females performed an interval running protocol in three menstrual cycle phases: early follicular phase (EFP), late follicular phase (LFP), and mid-luteal phase (MLP). The protocol consisted of 8 × 3-min bouts at 85% of their maximal aerobic speed (vVO2peak) with a 90-s rest between bouts and a final 5-min active recovery at 30% vVO2peak. All variables were averaged every 15 s, obtaining 19 moments during recovery (time factor). To analyze the effects of the menstrual cycle on the final active cardiorespiratory recovery, an ANOVA for repeated measures was performed. ANOVA showed an effect on menstrual cycle phase on ventilation (EFP: 1.27 ± 0.35; LFP: 1.19 ± 0.36; MLP: 1.27 ± 0.37), breathing frequency (EFP: 35.14 ± 7.14; LFP: 36.32 ± 7.11; MLP: 37.62 ± 7.23), and carbon dioxide production (EFP: 1120.46 ± 137.62; LFP: 1079.50 ± 129.57; MLP: 1148.78 ± 107.91). Regarding the interaction results (phase x time), ventilation is higher at many of the recovery times during the MLP, with less frequent differences between EFP and LFP (F = 1.586; *p* = 0.019), while breathing reserve is lower at many of the recovery times during MLP, with less time differences between EFP and LFP (F = 1.643; *p* = 0.013). It seems that the menstrual cycle affects post-exercise recovery specially during the MLP, rising ventilation and lowering breathing reserve, giving rise to an impaired ventilatory efficiency.

## 1. Introduction

The study of menstrual cycle influence on endurance exercise has recently increased to reach a better understanding of the effects of sex differences on physical exercise [1,2,3,4,5]. This is probably because recent studies have increased evidence that the response to physical exercise may depend on the sex of the participants.

However, although the relationship between post-exertion cardiorespiratory recovery, cardiorespiratory pathologies, and/or the risk of coronary heart disease is evident [6,7,8,9,10] and serves as a predictive tool for cardiovascular events [8,11,12,13,14], very little literature has been concerned about the study of cardiorespiratory recovery in sport population, and less considering women as participants. Interestingly, the available literature suggests a positive relationship between a greater cardiorespiratory recovery and sports performance [15,16].

The recovery period in other forms of exercise, such as high-intensity interval exercise, has been scarcely studied in comparison with the classic incremental running test [17], in which cardiac recovery exhibits a biphasic pattern with an initial rapid fall followed by a subsequent slow fall. The initial rapid fall is basically independent of the type of exercise, as it is more influenced by baroreflex sensitivity [18]. In contrast, the slow fall phase seems to be more dependent on the type of exercise [19]. There is evidence to suggest that the recovery response after exercise may differ between sexes, since substrate metabolism and circulatory homeostasis have shown differences in earlier studies [20]. Nonetheless, literature lacks studies focusing on this population. In this regard, some studies analyzing post-exercise recovery in both males and females reported results mixing both genders [21], while some others showing results by gender have a very small sample size (*n* = 3) [22]. Likewise, although studies analyzing sex hormone influence on females’ response to exercise has recently increased [1,2,3,4,5], females’ recovery throughout the menstrual cycle and the different phases remains unknown.

To date, there is no study that focuses on determining recovery after interval running exercise as a function of recovery time pattern and menstrual cycle phase in women. We hypothesize that sex hormones may influence women’s recovery after intense exercise. Therefore, the aim of the present work was to assess menstrual cycle influence on post-exercise recovery following a high intensity interval exercise in trained females.

## 2. Methods

### 2.1. Participants

Thirteen endurance-trained females with eumenorrheic cycles were recruited for this study (see Table 1 for participants’ characteristics). An eumenorrheic menstrual cycle was defined as a regularly occurring menstrual cycle ranging from 24 to 35 days in length [23]; the intrasubject menstrual cycle duration was 29.8 ± 2.3 days. Participants had 7.4 ± 5.3 years of endurance training experience, with a training volume of 296 ± 184 min per week during the 6 months prior to recruitment. Sample size estimation, participants’ inclusion and exclusion criteria, and full protocols were previously reported by our group [24]. Additionally, to be included in the present study, participants were required to perform the interval running protocol described below with no intensity adjustments. The Research Ethics Committee of the Universidad Politécnica de Madrid approved the project, and participants provided written informed consent. This study is registered on clinicaltrials.gov (ID: NCT04458662).

### 2.2. Menstrual Cycle Monitoring

Participants performed the main project protocol during the following menstrual cycle phases: early follicular phase (EFP), late follicular phase (LFP), and mid-luteal phase (MLP). Each participant’s menstrual cycle length was calculated by a gynecologist using the calendar-based method with the information of the last six menstrual cycles prior to joining the study [25]. Participants were scheduled on the dates indicated for the above menstrual cycle phases. The EFP was detected due to the onset of menses and scheduled one to four days later. The LFP was scheduled between 1 and 3 days before expected ovulation, and the MLP was between 5 and 9 days after confirmed ovulation, which was evaluated using a home ovulation kit (Ellatest, Alicante, Spain). A second morning mid-stream urine sample was collected each day from 3 to 5 days before the LFP protocol until luteinizing hormone (LH) surge detection, which allegedly occurs 14–26 h before ovulation [25]. Additionally, sex hormone verification was carried out to confirm the menstrual cycle phase and to exclude deficient luteal phases [25].

### 2.3. Study Design

Participants were required to be in the laboratory of exercise physiology of the Faculty of Physical Activity and Sport Sciences of Universidad Politécnica de Madrid four times. On the first occasion, they underwent a screening protocol during the EFP to confirm the inclusion/exclusion criteria, since the lowest levels of iron-related parameters have been reported during this phase [26]. Firstly, baseline blood samples were collected in a rested and fasted state. A complete blood count and biochemistry and hormonal analysis were performed to verify the conformity of iron markers with the inclusion criteria and to dismiss any illness, hormonal disorders, or menstrual cycle dysfunction. On the same day, body weight was measured with a scale (Lafayette Instruments Company, Lafayette, IN, USA) and height with a stadiometer (Holtain Limited, Crymych, UK), followed by a dual-energy X-ray absorptiometry (DXA) to determine body composition (Lunar Prodigy; GE Healthcare, Madison, WI, USA). Then, after a non-standardized meal and rest (a minimum of 2 h after feeding), participants performed a maximal ramp test to determine each participant’s peak oxygen consumption (VO_2peak_) and maximal aerobic speed (vVO_2peak_), as previously described [24]. In addition, a standardized spirometry test was performed prior to the maximal ramp test to measure the forced expiratory volume in 1 s (FEV_1_) and calculate breathing reserve.

After this screening day, participants reported to the laboratory to perform an interval running protocol during the mentioned menstrual cycle phases: EFP (day 3.6 ± 0.9), LFP (day 11.9 ± 1.9) and MLP (day 21.4 ± 2.3). In addition, the mean day of the positive result in the LH test was 13.4 ± 1.7. The order of these running protocols was randomized and counterbalanced by the main researcher to avoid a learning effect in our participants, which could affect our results. The following test orders were randomized, and in no case an order involved evaluating a volunteer in more than two menstrual cycles: EFP-LFP-MLP, LFP-MLP-EFP, MLP-EFP-LFP, LFP-EFP-MLP, and EFP-MLP-LFP.

**Figure 1:**
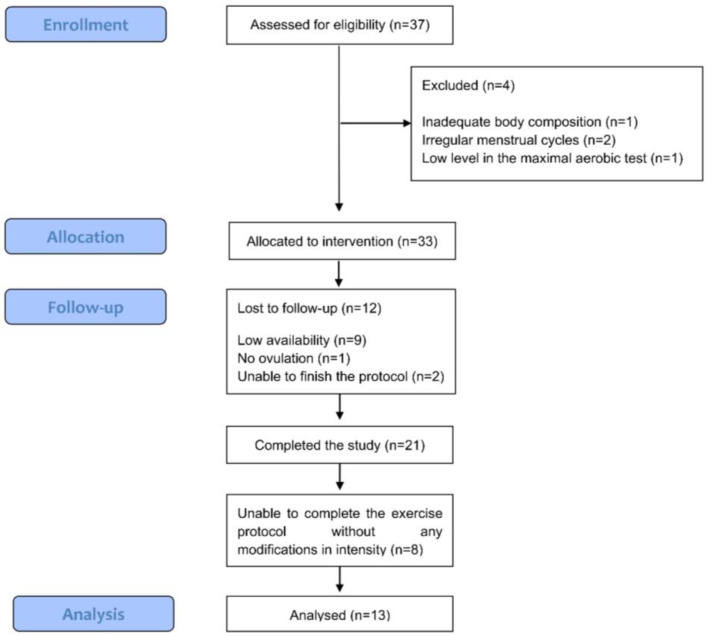
Flow diagram of study participants modified from Peinado et al. (2021) [24] according to STROBE guidelines.

Participants abstained from alcohol, caffeine, and any intense physical activity or sport 24 h before coming to the laboratory. Protocols were initiated between 8 a.m. and 10 a.m. and after the participants had breakfast at least 2 h earlier. Nutritional recommendations were provided to the participants by a nutritionist, and they were instructed to follow them 48 h prior to the protocol to avoid potential nutritional disturbances on the main outcomes analyzed. Furthermore, participants ate the same breakfast for each protocol during the different menstrual cycle phases. Firstly, a blood sample was collected before the running protocol to analyze 17β--estradiol, progesterone, LH, follicle-stimulating hormone (FSH), and prolactin. The handling and processing of the blood samples, as well as the methods of analysis and coefficients of variation reported by the laboratory, can be found in [24]. Subsequently, participants started the interval running protocol, which consisted of a 5-min warm-up at 60% of the vVO_2peak_, followed by 8 bouts of 3 min at 85% of the vVO_2peak_ with a 90-s recovery at 30% of the vVO_2peak_ between bouts, followed by a final 5-min cooling down period at 30% of the vVO_2peak_. Of the 21 participants who started the study, only 13 were able to complete the protocol as designed, 8 of them needing to slow down. Therefore, the latter were excluded and finally 13 participants were analyzed (Figure 1). During this protocol, ventilation, tidal volume (VT), breathing frequency (BF), oxygen consumption (VO_2_), carbon dioxide production (VCO_2_), respiratory exchange ratio (RER), ventilatory equivalent for VO_2_ (EqO_2)_ and ventilatory equivalent for VCO_2_ (EqCO_2_), and breathing reserve determined as (MVV − VE/MVV) × 100, when MVV is the maximal voluntary ventilation (MVV) and VE is submaximal ventilation measured during the recovery exercise period were measured. MVV was estimated using de Wasserman [27] equation MVV = 40 * FEV1. All variables were measured breath-by-breath with the gas analyzer Jaeger Oxycon Pro (Erich Jaeger, Viasys Healthcare, Friedberg, Germany) for which validity and reliability have been previously demonstrated [28,29]. In addition, heart rate (HR) was continuously recorded beat-to-beat using a HR monitor (RS400sd; Polar Electro Oy, Kempele, Finland).

To analyze the time pattern of the cooling down period, the cardiorespiratory variables were averaged every 15 s from second 0 of the cooling down to second 285, obtaining 19 time points.

### 2.4. Statistical Analysis

Data are presented as the mean ± standard deviation (SD) in tables and mean ± standard error of the mean (SEM) in figures. A Shapiro–Wilk test to assess the normality of the variables was conducted. An ANOVA for repeated measures (3 × 19) was used to analyze the effects of the menstrual cycle phase (EFP, LFP, and MLP) and the effects of the menstrual cycle phase depending on the moment (time) of the cooling down period on the aforementioned variables. Moreover, when Mauchly’s test indicated a violation of the assumption of sphericity, the Greenhouse–Geisser correction was applied. A Bonferroni post-hoc test was conducted where significant differences were found on the analyzed factors. Effect sizes for Bonferroni post-hoc comparisons were calculated using Cohen’s d to assess the magnitude of the effect on the changes found. Threshold values were set as small (≥0.2 and <0.5), moderate (≥0.5 and <0.8), and large (≥0.8). In addition, 95% confidence intervals (CI) were calculated. The statistical significance was set at *p* < 0.05. In addition, the effect size was considered meaningful when its CI did not include zero [30]. All procedures were conducted with SPSS software, version 25 (IBM Corp., Armonk, NY, USA).

## 3. Results

The characteristics of participants are show in Table 1.

Table 2 shows the concentration of sex hormones in the three studied phases of the menstrual cycle of the participants. This table shows that the values are in accordance with the variations established in the literature for the verification of the different phases of the menstrual cycle [23].

Table 3 shows the cardiorespiratory variables in the menstrual cycle phases. Differences were found for VT, BF, VCO_2_, EqO_2_, and EqCO_2_ in the menstrual cycle phase factor (see F, *p*, d, and CI values in Table 3), while for the variables VO_2_, RER, and HR no difference was found.

No interaction was found between menstrual cycle phase and recovery time (*p* > 0.05) for VT, BF, VO_2_, VCO_2_, RER, EqO_2_, EqCO_2,_ and HR, while an interaction between menstrual cycle phase and recovery time was observed for ventilation (F = 1.586; *p* = 0.019) and breathing reserve (F = 1.643; *p* = 0.013).

**Figure 2:**
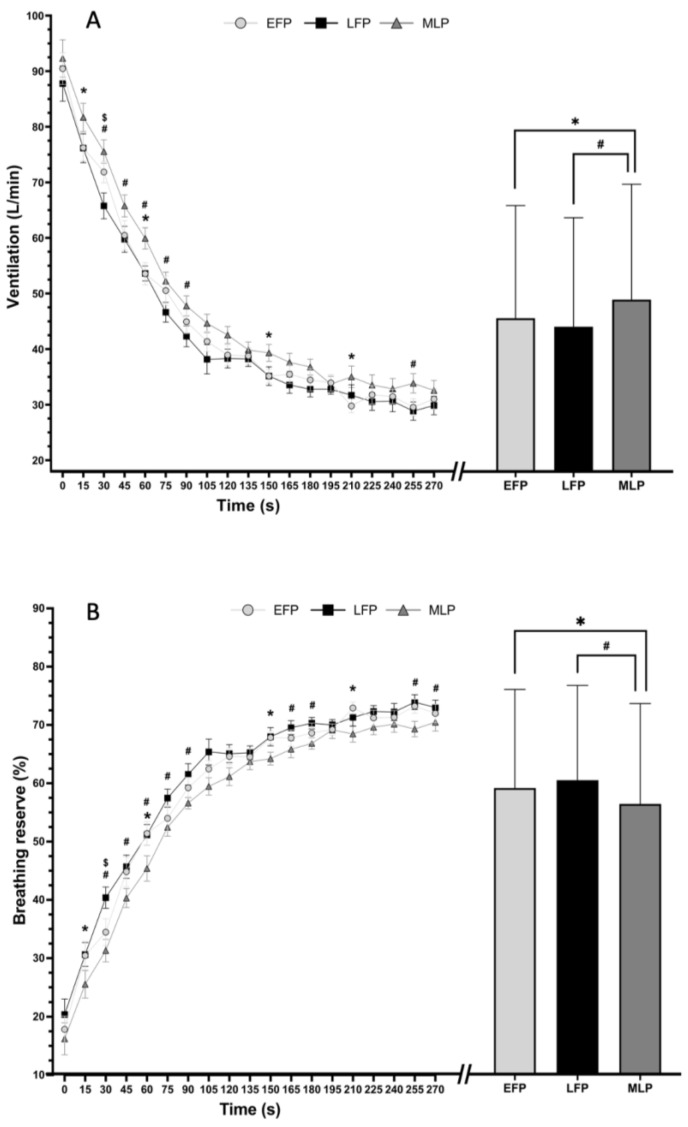
Ventilation (**A**) and breathing reserve (**B**) in the different phases of the menstrual cycle during the 5 min (0 s to 300 s) of cool down after an interval running session. Mean ± SEM values are show on the right side of the picture. * Differences between EFP vs. MLP (*p* < 0.05); # Differences between LFP vs. MLP (*p* < 0.05); $ Differences between EFP vs. LFP (*p* < 0.05). Abbreviations: EFP, early follicular phase; LFP, late follicular phase; MLP, mid-luteal phase.

Figure 2A shows the ventilation response. The right side of the figure shows the differences between phases. There are differences between the EFP and the MLP (d = 0.162; CI = −0.024 to 0.348), and also between the LFP and the MLP (d = 0.242; CI = 0.036 to 0.447). Regarding the interaction results, the left side of the figure shows that ventilation is higher at many of the recovery times during the MLP, with less frequent differences between EFP and LFP (see in the figure).

Figure 2B shows the breathing reserve. Pooling across all recovery times (right side of the figure), there are differences between the EFP and MLP (58.8 ± 16.8 and 56.1 ± 17.2 mean and SD, respectively, *p* = 0.028; d = −0.160; CI = −0.345 to 0.024). Differences were also found between LFP and MLP (60.2 ± 16.2 and 56.1 ± 17.2, *p* = 0.001; d = −0.244; CI = −0.449 to −0.039). No differences were observed between EFP and LFP phases, as *p* > 0.05. On the left side of the Figure 1B, the interaction between menstrual cycle phase and time is shown. It can be observed that the breathing reserve is lower at many of the recovery times during MLP, with less time differences between EFP and LFP (see *p* values in the figure).

## 4. Discussion

The aim of the present study was to observe the menstrual cycle influence on post-exercise recovery following high intensity interval exercise. Our work showed a menstrual cycle phase impact on VE and BR throughout the post-exercise recovery following a high intensity interval exercise. Specifically, our results exhibited higher VE along with lower breathing reserve in the MLP compared to the EFP and LFP (Figure 1A). Breathing reserve response on post-exercise recovery in women has not been previously described.

It is necessary to consider that BR uses an equation where the MVV of each subject is a constant calculated through FEV1, and variations occur through the change in VE during recovery. For this reason, the pathway of ventilation and ventilatory reserve are very similar, as shown in Figure 1A,B. However, there is a combination of conditions that could cause VE and BR to be dissimilar. In healthy men, maximal exercise is limited by cardiac output, and not by ventilatory reserve so that at the end of maximal exercise there is still respiratory reserve [31], but in pathology it is different. In individuals with pulmonary fibrosis, other respiratory restrictions or poor physical condition, the ventilatory reserve is compromised and therefore, both ventilation and respiratory reserve could be a handicap for physical performance [32].

A limitation of the present study is that the spirometry test was performed on a single occasion. Spirometry at each phase of the menstrual cycle would have allowed us to know whether FEV1, and thus MMV, are influenced by the menstrual cycle. In any case, it can be observed that after 5 min of recovery, the participants in this study are far from being recovered from the ventilatory point of view (see Figure 1B) and may need up to 20 min to reach resting values [33].

Considering the components of this BR, VE outcomes from the present study are supported by previous research, which observed a rise in VE during the MLP when running [2,4,34]. Authors from these studies agree in the fact that increments in respiratory variables occur during the MLP due to progesterone’s peak in this phase. There is a strong basis of evidence that high levels of progesterone enhance the chemosensitivity of the hypothalamus chemoreceptors, lowering the threshold of the medullary respiratory center, and this in turn increases VE [35,36]. Likewise, previous results from our research group [2] have shown an influence of menstrual cycle phase on ventilatory response throughout the cool down (*p* = 0.007), with a mild rise of ventilation during the MLP (45.9 ± 6.0 L/min) in comparison to the LFP (42.6 ± 6.1 L/min) and the EFP (43.2 ± 6.4). These post-hoc comparisons were not significant, which might be explained by the fact that some volunteers from the Rael et al. [2] study required intensity adjustments to finish the exercise protocol. The latter may suggest that intensity was supramaximal for these participants, and this might have masked menstrual cycle phase effects in recovery.

Concerning the breathing pattern, some differences were observed during the three menstrual cycle points studied. The MLP and EFP showed higher VT compared to the LFP, while the BF during the MLP was higher in contrast to the EFP. Both responses could explain the higher VE observed during the MLP. The increase in VE and inspiratory flow may indicate the influence of progesterone in stimulating the respiratory drive, either centrally as explained above, or peripherally, or by both [37]. Another possible reason to explain the higher VT and BF during the MLP is the influence of ovarian hormones on the contractile component of respiratory muscles [38]. Reinforcing this idea, the positive correlations observed between estrogen, progesterone, and some ventilatory parameters suggest an influence of these hormones on the strength of the thoracic muscles during the luteal phase [39]. However, overall body-strength-related measures seem to be minimally altered by sex hormone fluctuations during the menstrual cycle [40].

Regarding ventilatory equivalents, EqCO_2_ values were higher in the MLP, which is supported by previous studies [5]. The EqCO_2_ during exercise reflects ventilatory efficiency and evaluates VE relative to metabolic demand [41]. Our results suggest a reduced ventilatory efficiency during active recovery in the MLP (Table 3). However, this finding should not be related to poorer cardiovascular response, since a previous study showed no differences among the menstrual cycle phases throughout the same interval running protocol [2]. In fact, a recent meta-analysis revealed no differences in exercise performance variables between menstrual cycle phases, except a trivial performance decrease in the EFP [1]. Since at the end of 5 min all subjects reached the same ventilatory values of recovery, future studies are needed to elucidate why in the MLP these values are lower than in other phases, and whether this fact may compromise the practice of exercise at the ventilatory level with recoveries shorter than 5 min.

Finally, the main limitation of this study is the elevated inter-subject hormonal variation. In our study, estrogen values in the LFP ranged from 135.53 to 293.84 pg/mL, thus making it difficult to clarify the influence of sex hormones on any exercise response. Moreover, the intra-subject variation should be also considered when analyzing successive cycles.

## 5. Conclusions

In conclusion, from a ventilatory point of view, the mid-luteal phase is the time in the menstrual cycle when recovery after high-intensity exercise might be delayed. This finding could be useful for training prescription in recreational athletes. However, given the large heterogeneity found in our participants, our results should be interpreted with caution, as each woman’s individual response may differ from these findings. Further studies are needed to investigate post-exercise recovery in females in order to determine how the phases of the menstrual cycle may affect both recovery and cardiorespiratory performance in subsequent exercise.

## Figures and Tables

**Table 1:** Characteristics of the participants (*n* = 13).

	Mean	SD	95% CI
Age (y)	30.8	5.9	27.6 to 34.0
Height (m)	1.65	0.06	1.62 to 1.68
Weight (kg)	61.0	8.4	56.4 to 65.6
BMI (kg/m^2^)	21.7	2.1	20.6 to 22.8
BMD (g/cm^2^)	1.205	0.087	1.157 to 1.252
Fat mass (kg)	16.1	6.2	12.7 to 19.4
Fat mass (%)	26.6	7.8	22.4 to 30.8
Lean mass (kg)	42.2	3.6	40.3 to 44.2
Lean mass (%)	71.9	8.9	67.1 to 76.8
VO_2_ max (mL/kg/min)	48.2	5.1	45.4 to 51.0

BMI: body mass index; BMD: bone mass density; VO_2_ max: maximal oxygen consumption; SD: standard deviation; CI: confidence interval.

**Table 2:** Concentration of sex hormones in the three phases of the menstrual cycle.

	Early Follicular Phase (EFP)			Late Follicular Phase (LFP)			Mid-Luteal Phase (MLP)		
	Mean	SD	95% CI	Mean	SD	95% CI	Mean	SD	95% CI
Estrogen (pg/mL)	40.94	28.67	28.68 to 53.21	214.69	185.06	135.53 to 293.84	142.20	89.14	104.07 to 180.32
Progesterone (ng/mL)	0.34	0.19	0.27 to 0.42	0.38	0.31	0.25 to 0.52	11.46	5.08	9.29 to 13.63
LH (mIU/mL)	6.66	1.61	5.97 to 7.34	13.60	8.01	10.18 to 17.03	6.03	2.77	4.84 to 7.21
FSH (mIU/mL)	8.52	4.50	6.59 to 10.44	6.70	3.15	5.35 to 8.05	3.62	1.41	3.02 to 4.23
Prolactin (mIU/L)	512.67	352.92	361.72 to 663.61	440.47	144.43	378.69 to 502.24	553.85	262.79	441.45 to 666.24

LH: luteinizing hormone; FSH: follicle-stimulating hormone; SD: standard deviation; CI: confidence interval.

**Table 3:** Results of ANOVA for repeated measures for phase factor in the cardiorespiratory variables (*n* = 13).

	Early Follicular Phase (EFP)			Late Follicular Phase (LFP)			Mid-Luteal Phase (MLP)			F-Value	*p*-Value
	Mean	SD	95% CI	Mean	SD	95% CI	Mean	SD	95% CI		
VT (L/min)	1.27	0.35	1.16 to 1.38	1.19 ^a^	0.36	1.09 to 1.29	1.27 ^b^	0.37	1.16 to 1.38	7.592	0.003
BF (1/min)	35.14	7.14	32.71 to 37.57	36.32	7.11	33.70 to 38.94	37.62 ^c^	7.23	34.86 to 40.38	8.319	0.002
VO_2_ (mL/min)	1179.15	163.17	1075.48 to 1282.83	1123.09	169.62	1015.32 to 1230.86	1163.71	164.84	1058.97 to 1268.44	1.376	0.273
VCO_2_ (mL/min)	1120.46	137.62	1033.02 to 1207.89	1079.50	129.57	997.18 to 1161.82	1145.78 ^d^	107.91	1077.21 to 1214.34	4.701	0.02
RER	0.94	0.05	0.91 to 0.97	0.96	0.09	0.90 to 1.02	0.99	0.10	0.92 to 1.05	0.948	0.403
EqO_2_	38.18	4.70	35.19 to 41.16	38.96	4.40	36.16 to 41.76	42.21	5.93	38.45 to 45.98	5.168	0.014
EqCO_2_	40.49	3.85	38.04 to 42.93	40.78	3.50	38.55 to 43.00	42.77 ^ef^	3.58	40.50 to 45.05	8.377	0.002
HR (bpm)	139.42	14.11	130.54 to 148.30	139.69	14.41	130.62 to 148.75	138.48	12.27	130.77 to 146.20	0.194	0.743

^a^ Differences between EFP vs. LFP (*p* = 0.011; d = 0.22); ^b^ Differences between LFP vs. MLP (*p* = 0.003; d = −0.22); ^c^ Differences between EFP vs. MLP (*p* = 0.007; d = −0.35); ^d^ Differences between LFP vs. MLP (*p* = 0.018; d = −0.56); ^e^ Differences between EFP vs. MLP (*p* = 0.010; d = −0.62); ^f^ Differences between LFP vs. MLP (*p* = 0.014; d = −0.56). VT: ventilatory threshold; BF: breathing frequency; VO_2_: oxygen consumption; VCO_2_: carbon dioxide production; RER: respiratory exchange ratio; EqO_2_: ventilatory equivalent for VO_2_; EqCO_2_: ventilatory equivalent for VCO_2_; HR: heart rate.

## Data Availability

The data presented in this study are available on request from the corresponding author. The data are not publicly available due to internal policy reasons.
